# Magnetic Sensing with Ferrofluid and Fiber Optic Connectors

**DOI:** 10.3390/s140303891

**Published:** 2014-02-25

**Authors:** Daniel Homa, Gary Pickrell

**Affiliations:** Department of Materials Science and Engineering, Virginia Tech, Blacksburg, VA 24061, USA; E-Mail: pickrell@vt.edu

**Keywords:** optical fiber, fiber optic sensor, magnetic sensor, magnetic fluids, ferrofluid, nanotechnology, electrical field sensor

## Abstract

A simple, cost effective and sensitive fiber optic magnetic sensor fabricated with ferrofluid and commercially available fiber optic components is described in this paper. The system uses a ferrofluid infiltrated extrinsic Fabry-Perot interferometer (EFPI) interrogated with an infrared wavelength spectrometer to measure magnetic flux density. The entire sensing system was developed with commercially available components so it can be easily and economically reproduced in large quantities. The device was tested with two different ferrofluid types over a range of magnetic flux densities to verify performance. The sensors readily detected magnetic flux densities in the range of 0.5 mT to 12.0 mT with measurement sensitivities in the range of 0.3 to 2.3 nm/mT depending on ferrofluid type. Assuming a conservative wavelength resolution of 0.1 nm for state of the art EFPI detection abilities, the estimated achievable measurement resolution is on the order 0.04 mT. The inherent small size and basic structure complimented with the fabrication ease make it well-suited for a wide array of research, industrial, educational and military applications.

## 1. Introduction

Magnetometers are used in a very diverse range of applications, from locating submarines and sunken ships to heart monitors and sensors in anti-locking brakes. The ability to accurately detect and sense magnetic fields continues to enable the advance of technologies that support our ever changing lifestyles and daily lives.

Magnetic sensors based on electrical devices are often sensitive to environmental conditions such as electric fields and temperature. The most sensitive magnetic sensors are within the class of Superconducting Quantum Interference Devices (SQUIDs), and are limited due to the cryogenic requirements, enormous size, expense and sensitivity to environmental conditions. Fiber optic sensors are typically much smaller and often rely on relatively mature technologies. Several different optical fiber magnetic sensors have been introduced over the past several decades, but are often difficult to manufacture, and lack the reliability required for the desired applications.

Recently, fiber optic magnetometers have been demonstrated via the interrogation of commercially available ferrofluids with common optical fiber sensing schemes, as shown elsewhere [1–5]. The nanosuspensions exhibit a strong magnetic response while retaining their characteristics under the influence of a strong magnetic field. Several researchers have incorporated the ferrofluid into the holes of a photonic crystal fiber or glass capillary tubes, and more details can be found in published work [6–11]. Although promising, the sensor designs are often extremely cumbersome to fabricate, and require expensive and exotic equipment. Presented in this letter is a simple and cost effective extrinsic Fabry Perot interferometer (EFPI) has been demonstrated that can readily detect magnetic flux densities in the range of 0.5 mT to 12 mT.

## 2. Experimental Section

### 2.1. Basic Sensor Configuration

**Figure 1.**
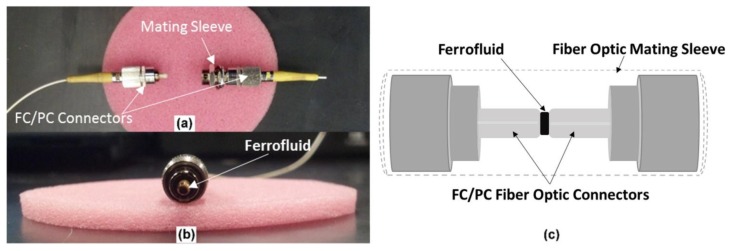
(**a**) Assembly components for the magnetic sensor: Fiber optic jumper cable with FC/PC end connector, FC/PC union, and terminated FC/PC connector. (**b**) A “drop” (<10 μL) of ferrofluid on the end face of on input FC/PC connector end face. (**c**) Schematic of the assembled EFPI sensor.

The basic concept takes advantage of the air gap between two flat optical connectors upon “improper” installation of a fiber optic mating sleeve, as seen in Figure 1a. In most applications, the air gap is undesirable because it results in an increased attenuation across optical fiber transmission links. The air gap can also result in Fresnel reflections at the connector end—air interface producing optical fringe patterns; an excellent template for an EFPI sensor.

An EFPI was fabricated by inserting two FC/PC optical fiber connectors into a standard mating sleeve while allowing an air gap to exist between the fiber end faces. Prior to insertion into the matting sleeve, a “drop” of ferrofluid (<10 μL) was placed on the “reflector” or “transmission” FC/PC, as shown in Figure 1b. The sensor was manually assembled while monitoring the air gap distance with a commercial optical interrogator; a Micron Optics sm125 unit. The mating sleeves properly aligned the cores of the connectorized fibers at air gap distances in the range of 70–300 microns.

Sensors were constructed with two different Ferrotec ferrofluid types as seen in Table 1. The ferrofluids are light hydrocarbon oils that carry iron oxide nanoparticles (diameters of 10 nm). The EMG900 nanosuspension has a particle concentration that is more than double than that of the EFHI suspension. The increased concentration results in a ferrofluid with a viscosity, saturation magnetization, and initial magnetic susceptibility that is larger than the EFHI nanosuspension.

### 2.2. Experimental Design

**Figure 2.**
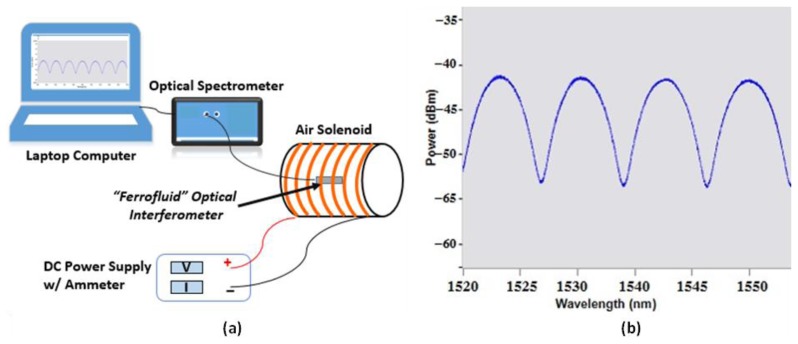
(**a**) Test setup with air solenoid and (**b**) EFPI fringe pattern.

The sensor response to magnetic flux densities over the range of 0 to 12 mT was monitored with a commercially available air solenoid purchased from Science First^®^. The sensors were positioned inside the air solenoid and the magnetic flux density was varied by changing the electrical current delivered to the copper windings, as shown in Figure 2a. The sensor was interrogated with a Micron Optics unit, SM125. The positions of selected fringe pattern peaks were monitored with the Enlight software interface provided with the unit, as shown in Figure 2b.

The magnetic field strength generated by the air solenoid was verified with measurements obtained from a more traditional device. A Lutron Milli-Gauss Meter, GU-3001, was employed to verify the performance of the fiber optic magnetic sensors. It has a measurement range of −3,000 mG to 3,000 mG, DC/AC- 300.0 μT to 300.0 μT, DC/AC and a resolution of 1 mG/0.1 μT.

## 3. Results and Discussion

**Figure 3.**
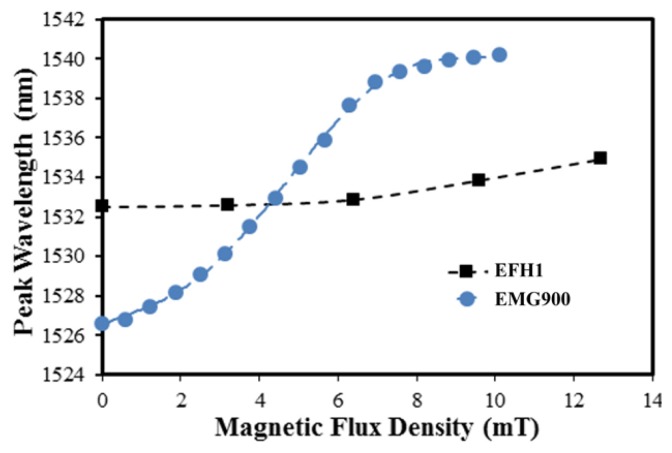
The response of a magnetic fiber optic sensor with EFHI and EMG900 ferrofluids over a magnetic flux density range of 0 to 12 mT.

The magnetic flux density response of both sensor types can be seen in Figure 3. As expected, an increase in iron oxide nanoparticle concentration improved the sensitivity of the sensor; the EMG900 ferrofluid demonstrated a sensitivity of approximately 2.3 nm/mT. The sensor with the EFH1 ferrofluid maintained a sensitivity of 326 pm/mT in the region of linear response, which is consistent with previously reported values for a similar sensor design employing photonic crystal fiber [11]. The magnetic fluid concentration of the EFH1 was specified as 7.9% by volume, while the EMG900 was 17.7 vol.%. This 125% increase in particle concentration yielded almost an order of magnitude improvement in the sensitivity upon exposure to a magnetic field. Furthermore, the range of linear response was extended in the EMG900 based sensor which is commensurate with the increase in initial magnetic susceptibility and saturation magnetism of the ferrofluid. To the best of our knowledge, the magnetic flux density sensitivity of the EMG900 based EFPI sensor is the greatest demonstrated in a fiber optic sensor using magnetic fluids.

The methods of optical interrogation, data acquisition, and the processing and analysis techniques employed to interpret the data can have a profound effect on the measurement quality. As mentioned earlier, we used wavelength peak tracking software to evaluate the resolution and/or sensitivity of the EFPI sensor system to magnetic fields. Many more appropriate interrogation and analysis techniques for EFPI based sensors are well documented in the literature and readily available for implementation with our fiber optic sensor [12–16]. Assuming a resolution of 1 pm with the state of the art EFPI signal processing techniques, the basic sensor configuration could yield a measurement resolution in the range of 0.04 mT. Furthermore, the initial experimentation performed in this study suggests that a direct understanding of the effect of the magnetic fluid viscosity and composition could result in further improvement in performance.

## 4. Conclusions/Outlook

The experimental results demonstrate the ability to accurately detect magnetic fields with an economically and mechanically robust design. The methods of optical interrogation, data acquisition, processing and analysis employed to interpret the data can have a profound effect on the measurement quality. The sensor resolution can be improved by the use of more appropriate EFPI sensor interrogation and analysis techniques that are readily available for implementation with the discussed fiber optic sensor [12–16]. In addition, the basic sensor components and assembly procedures can be readily optimized to improve overall measurement performance and stability. Subsequently, tuning of the sensor performance via the magnetic fluid composition will pave the way for applications in consumable and deployable magnetic sensors, as well as a potential alternative to SQUID devices.

## Figures and Tables

**Table 1.** Ferrofluid Material Properties.

**Property**	**EFH1**	**EMG900**
Carrier Fluid	Hydrocarbon	Hydrocarbon
Particle Diameter (nm)	10	10
Particle Concentration (vol. %)	7.9	17.7
Saturation Magnetization (mT)	44	99
Viscosity (mPa-s)	6	60
Initial Magnetic Susceptibility	2.64	18.6

nm = nanometer, vol.% = volume percentage, mPa-s = millipascal second.
